# Temporal and Spatial Dynamics of *Vibrio harveyi*: An Environmental Parameter Correlation Investigation in a 4-Metre-Deep *Dicentrarchus labrax* Aquaculture Tank

**DOI:** 10.3390/microorganisms12061104

**Published:** 2024-05-29

**Authors:** Alix Da Fonseca Ferreira, Roxane Roquigny, Thierry Grard, Cédric Le Bris

**Affiliations:** Université du Littoral Côte d’Opale, UMRt 1158 BioEcoAgro, USC ANSES, INRAe, Université d’Artois, Université de Lille, Université Picardie Jules Verne, Université de Liège, Junia, 62200 Boulogne-sur-Mer, France; alix.da-fonseca-ferreira@univ-littoral.fr (A.D.F.F.); roxane.roquigny@univ-littoral.fr (R.R.); thierry.grard@univ-littoral.fr (T.G.)

**Keywords:** aquaculture, vibriosis, *Vibrio harveyi*, biofilms, planktonic, environment, spatio-temporal study

## Abstract

Nowadays, European seabass (*Dicentrarchus labrax*) aquaculture is undergoing a significant expansion. Nevertheless, the aquaculture industry is plagued by vibriosis. The spatial and temporal dynamics of *Vibrio harveyi* were studied on a European seabass farm in northern France during seven months of 2022. Concrete specimens were suspended and water was pumped from different depths (0.3 m, 2.15 m and 4 m deep), providing insights into the biofilm and planktonic *V. harveyi* dynamics. The abundances of *V. harveyi*, in the biofilm and free-living forms, were positively correlated. The water parameters revealed seasonal fluctuations in temperature, pH, and salinity, with no significant differences observed across the water column. Quantification of *V. harveyi* revealed no significant differences between depths, but seasonality, with peak abundances observed in August, correlated with temperature increases. Principal component analysis identified temperature as a primary driver, but also additional parameters, such as salinity and pH. Vibriosis occurred during the sampling period, providing valuable insights into the conditions before, during, and after the outbreaks. This study underscores the importance of understanding *V. harveyi* behaviour in aquaculture, particularly in the context of global warming, for effective disease management and sustainable practices.

## 1. Introduction

Given the swift expansion of the global population and the sharp decrease in catches from marine fisheries, the advancement of aquaculture has gained substantial attention as a means to guarantee worldwide food supplies [1]. The European seabass (*Dicentrarchus labrax*) stands out as one of the most economically significant fish species harvested in Europe. Over recent decades, the European seabass aquaculture sector has experienced robust growth. Overall production has reached 80,786 tonnes, amounting to a value of EUR 470 million in 2020 [2]. However, like many other species, *D. labrax* is susceptible to various bacterial diseases, including vibriosis (*Vibrio* spp.). These diseases pose significant threats to the seabass aquaculture sector [3]. They not only cause mass mortality events but also have substantial economic consequences [4,5].

Throughout the whole production chain, vibriosis stands out as the most significant threat to seabass. It is characterised by various symptoms in seabass, including anorexia, erratic swimming behaviour, exophthalmia, darkening of body colour, pale gills, haemorrhage, ulcers, and skin lesions, and it can be fatal [6,7,8]. It can be caused by various species of *Vibrio*, such as *Vibrio harveyi*, which has recently emerged as a significant concern in seabass aquaculture [9,10,11].

*Vibrio harveyi* thrives in natural marine ecosystems. Its ability to act as a pathogen in a wide range of cultivated hosts, such as crustaceans, molluscs, and fish, is a concern, notably for open-circuit aquaculture farms [6]. Importantly, *V. harveyi* exhibits various states of existence, including free-swimming and within biofilms attached to both living and non-living surfaces, or engaged in symbiotic and host–pathogen interactions [12]. The formation of biofilms on tank walls can significantly heighten the risk of vibriosis outbreaks. In addition, outbreaks of vibriosis in seabass farms exhibit pronounced seasonality, occurring more frequently when water temperatures reach their maximum [13]. Increasing temperatures, particularly with global warming, may boost the abundance and possibly the virulence of viable *V. harveyi* in the surrounding environment, consequently increasing the presence of *V. harveyi* and its damage in tanks. Actually, the occurrence of vibriosis episodes remains unpredictable despite the high temperatures, as highlighted by Mougin et al. [14]. In their study, conducted in a small tank (75 m^3^), no instances of vibriosis occurred during the 7-month sampling campaign, despite the clear impact of temperature on the *V. harveyi* concentrations. The study was conducted in a 1.3-metre-deep tank, which is not deep enough to observe the heterogeneity of environmental parameters and their potential impact on *V. harveyi* and vibriosis development. This underscores the need for further investigations into the relationships between environmental parameters, *V. harveyi* concentrations and dynamics, and vibriosis outbreaks. Additionally, to gain a more comprehensive understanding of these relationships, studies should expand to larger tanks, where environmental heterogeneity is more likely, allowing for a more nuanced examination of repartition within the water column. Importantly, biofilms add an additional layer of complexity to the understanding of vibriosis development. In biofilms, interspecific interactions are facilitated, and previous research conducted by Roquigny et al. [15] has already highlighted distinctions in microbial communities and their temporal shifts in an aquaculture tank, which may interact with *V. harveyi*. In this context, the first question that arises is whether the temporal and spatial dynamics of these two forms of *V. harveyi*—free-swimming and within biofilms—are interconnected, and if one compartment of the tank acts as a reservoir for each form. Before understanding the development of vibriosis outbreaks, it is thus essential to comprehend *V. harveyi’s* spatial and temporal distribution within aquaculture tanks, as well as the environmental conditions that favour its proliferation and persistence. Therefore, through a comprehensive investigation conducted from May to November 2022, encompassing sampling periods before, during, and after episodes of vibriosis outbreaks, we sought to elucidate the spatial persistence of *V. harveyi* in water and biofilms, particularly during “heatwaves” or vibriosis outbreaks. This sampling was performed through both biofilm and water sampling, and environmental factors were measured alongside. Moreover, the conducted sampling was performed at three different depths within the water column in a four-metre deep tank to capture potential variations across the vertical gradient. This study aimed to identify potential environmental niches within these tanks where *V. harveyi* may thrive and persist. The objective was to conduct a diagnostic assessment of the tank environment and gain a deeper understanding of the pathogen–environment interaction that could lead to vibriosis outbreaks. It could lay the groundwork necessary to effectively address its virulence and mitigate the risk of vibriosis in aquaculture settings.

## 2. Materials and Methods

### 2.1. Sampling Site and Rearing Conditions

From May to November 2022, a sampling campaign was conducted at Aquanord-Ichtus, an aquaculture establishment situated in Gravelines (France), along the coast of the North Sea. This facility is strategically located near a nuclear power plant, which serves as a source of warm water for the flow-through tank systems via its reactor water cooling system. The facility benefits from thermoregulated water throughout the year, except in summer when the facility faces challenges in cooling the water tank more than the environmental water.

The cubical aquaculture tank chosen is an outdoor facility with a volumetric capacity of approximately 975 cubic m, a height of 4 m, and a capacity of up to 68,000 seabass. The average fish stock density within the tank was 32 kg per cubic meter at the beginning of the campaign, and the average individual fish weight was 517 g.

The fish were fed once or twice daily based on the total biomass in the system, following a theoretical feeding table. Oxygenation was performed to maintain optimal dissolved oxygen (DO) levels, which should range between 3 and 6.5 mg·L^−1^. To overcome the absence of vaccines against *V. harveyi*, Aquanord-Ichtus has started to create self-vaccines. This approach enables farmers to use oxytetracycline, an antibiotic, only in extreme cases when vibriosis-induced mortalities are exceptionally high and not effectively managed by other zootechnical measures. Vibriosis diagnosis was determined by a veterinary laboratory collaborating with the fish farm, through bacterial cultures and strain identifications from moribund fish. During vibriosis outbreaks and elevated fish mortality on the farm, the farmers administered oxytetracycline treatment once a day in the food for 7–10 days, ensuring antibiotic coverage for about 4 weeks.

### 2.2. Sample Collection and Environmental Parameters

To study the spatial and temporal dynamics of *V. harveyi* in biofilms, cylindrical concrete specimens (5 cm × 2.5 cm) constructed from the same material as the tank walls, were attached to a rope at three equidistant depths from the surface of the tank (top: 0.3 m deep, middle: 2.15 m deep, and bottom: 4 m deep). At each sampling time, five replicates of concrete were sampled at each depth. To study planktonic *V. harveyi*, alongside the concrete samples, 1 L of water in five replicates was also collected at each depth using a submersible pump. This process yielded 16 independent sampling times, and samples were taken from 16 ropes at the wall of the tank. The sampling apparatus was installed when the tank was in an empty state, and the initial sampling was performed one day after impoundment of the tank when the fish were added.

Biofilm and planktonic samples were collected every three weeks. Throughout the sampling campaign, the environmental seawater temperatures were carefully monitored, based on data provided by the MAREL Carnot buoy consulted from May to November 2022 [16]. This buoy supplied daily insights into the environmental temperatures of water used to partially supply the tanks. When the seawater temperatures exceeded 19 °C for two consecutive nights, sampling events were scheduled within a span of 4–5 days to anticipate the onset of vibriosis episodes. Given the sustained temperatures above 20 °C from mid-July to the end of August, a weekly sampling regimen was adopted, amounting to 7 samples (Table S1). At each sampling time, abiotic parameters such as the water temperature, pH, salinity, turbidity, DO, and conductivity were assessed within the tank at each layer, using an HI-9892 multi-parameter probe (Hanna Instruments, Leighton Buzzard, UK). Throughout the sampling campaign, the aquaculture facility supplied weekly data concerning the biomass and mortality rates for the studied rearing tank. A total of 240 water samples and 240 biofilm samples were collected during the sampling campaign.

### 2.3. Bacterial Isolation

The experimental protocol, established for a previous study at Aquanord-Ichtus [17], was replicated with minor adaptations. Briefly, we gathered five replicates of 1 L seawater samples from aquaculture tanks at different depths and sampling times. These samples were promptly chilled on ice during transportation to the laboratory and processed within a 3 h window following the sampling. Filtration involved the sequential use of 0.45 μm- and 0.22 μm-pore-size nitrocellulose filters (Grosseron SAS, Couëron, France), with the deployment of multiple filters when necessary to prevent clogging due to sample aggregation. Following this, the filters were immersed in a solution consisting of 30 mL of Luria–Bertani broth supplemented with 20% NaCl (LBS) containing 20% glycerol and stored at −80 °C until further analysis.

Concrete specimens were submerged at various depths: 0.3, 2.15, and 4 m. Post-collection, the specimens were individually collected and preserved in sterile physiological water and put on ice during transportation to the laboratory. They were immediately processed within a 2 h timeframe upon arrival. The biofilm collection protocol from the concrete, following Mougin et al.’s [17] guidelines with slight adaptations, used a systematic swabbing methodology after rinsing the blocks with sterile physiological water, targeting a specific surface area of 2.34 cm^2^. This process involved the use of a sterile, stainless steel jig and two consecutive sets of swabs (AB300250, Labomoderne, Gennevilliers, France). Subsequently, the swabs were immersed in LBS enriched with 20% glycerol and stored at −80 °C until further analysis.

To begin the analytical phases, all the samples were thawed and subjected to a 3 min vortexing process. After centrifugation at 8000× *g* for 10 min at room temperature, the resulting cell pellet was resuspended in physiological water. For subsequent steps, the samples were divided into two subsamples, with one of them (1800 μL) to be used for DNA extraction and *V. harveyi* quantification through real-time PCR, and the other (200 µL) kept for another study.

### 2.4. Quantification of V. harveyi Using Real-Time PCR

The 1800 μL subsamples underwent a centrifugation step at 8000× *g* for 10 min. Subsequently, a DNeasy PowerBiofilm kit (QIAGEN, Hilden, Germany) was used for total DNA extraction from the cell pellet, adhering to the manufacturer’s instructions. This method was chosen based on the procedures described by Mougin et al. [17]. This involved the use of *V. harveyi*-specific primers designed to target the *mreB* gene, which is present in *V. harveyi*’s genome as a single copy. The primers were synthesised by TIB MOLBIOL Syntheselabor GmbH (Berlin, Germany). To establish standard curves for the quantification, a series of 10-fold dilutions was prepared using pure DNA from the collection strain *V. harveyi* LMG 4044. For each individual assay, calibration curves were generated by plotting the mean threshold cycle (CT) values derived from two replicate series against the logarithm of the respective dilution factors. To mitigate potential interference from real-time PCR inhibitors, each extracted DNA template was analysed after being diluted ten-fold. The genome copy equivalent (GE) concentrations were calculated, taking the dilution factor into consideration. All the quantitative PCR reactions were conducted using a LightCycler^®^ 480 instrument (Roche Diagnostics, Meylan, France). These assays included a positive control (*V. harveyi* LMG 4044 DNA), a negative control (*V. campbellii* CIP 70.67 DNA), and a no-template control (NTC) devoid of DNA. To confirm the specificity of the fluorescent signals, melting curve analysis was performed. To ensure the reliability of the measurements, each extracted DNA template underwent two rounds of analysis.

### 2.5. Statistical Analysis

The data normality was assessed using either the Shapiro–Wilk test or the Kruskal–Wallis test, depending on the dataset distribution. Subsequently, the homogeneity of the variances was evaluated using Bartlett’s test. To determine significant differences among the variables across different layers, a one-way analysis of variance (ANOVA) was conducted. Post hoc comparisons were carried out using the least significant difference (LSD) method with the Bonferroni-adjusted *p*-values. In instances where the data did not meet the assumption of normality, the Kruskal–Wallis test was employed as a non-parametric alternative for comparing the medians among different groups. Additionally, a principal component analysis (PCA) was performed on the environmental parameters (temperature, pH, salinity, DO, and turbidity), *V. harveyi* water concentration, *V. harveyi* concrete concentration, and fish mortality. Before conducting the PCA, the raw data were standardised to ensure the equal weighting of each variable. The relationships among the variables were explored using a correlation matrix, employing the Spearman method. Prior to this analysis, the pairwise linear relationships were assessed to ensure the absence of linearity. All the statistical analyses, including the post hoc procedures, were conducted using R version 4.3.2 and R Studio version 2021.09.0 [18,19] with the “agricolae” [20], “factomineR” [21], “factoextra” [22], and “corrplot” [23] packages. All the graphics were drawn using the “ggplot2” package version 3.5.1 [24].

## 3. Results

### 3.1. Water Quality and Fish Mortality

The investigation involved a comprehensive examination of the water parameters in the top, middle, and bottom layers over the course of May to November 2022. Additionally, fish mortality data were collected and analysed. These data are summarised in Table 1. No significant differences were found across the water column for each sampling time for the recorded parameters, indicating the homogeneity of the environmental conditions in the tank. Nevertheless, some parameters showed variations throughout the sampling period. For instance, the mean temperature values ranged from a minimum of 16.70 °C in November to a maximum of 22.06 °C in August, demonstrating seasonal fluctuation. The pH values gradually decreased from an average of 8.07 in May to 6.87 in November. The values also showed increasing acidity with the depth in the water column. The DO concentrations remained constant. The fish mortality percentages were higher in July and highest in August, when the peak temperature values were recorded. The fish mortality episodes in May were likely associated with stress induced by the transfer of the fish into the tank.

### 3.2. Quantification of V. harveyi

**Figure 1:**
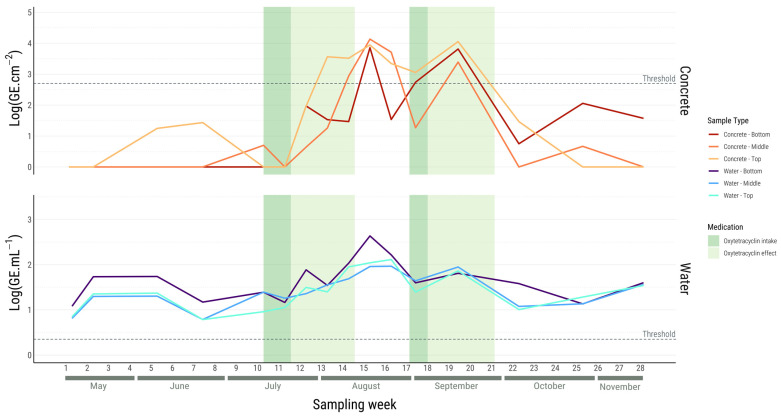
Biofilm concentrations of *V. harveyi* in log genome copy equivalents (GE)·cm^−2^ and *V. harveyi* concentrations in log GE·mL^−1^ according to the tank depth and time. The coloured lines correspond to the averages of the 5 replicates (raw concentration data are provided in Table S2). Antibiotic treatments with oxytetracycline are indicated in dark green, and their period of effect is estimated in light green. The dashed line marks the qPCR detection threshold for *V. harveyi* quantification, delineating the limit above which presence is reliably detected.

The abundance of *V. harveyi* was determined using *mreB*-targeted real-time PCR across the different layers for all the samples, as shown in Figure 1. The qPCR method has a specified threshold of detection in GE for each sample type: 2.24 GE·mL^−1^ (0.35 log(GE·mL^−1^)) for water samples and 501.19 GE·cm^−2^ (2.70 log(GE·cm^−2^)) for concrete samples. Despite the potential presence of *V. harveyi*, any concentration below this threshold was considered to be zero in the analysis (Table S2). For a given sampling time, no significant differences were found trough the water column for each sample type.

In the water samples, the presence of *V. harveyi* started to be detected from the beginning of the sampling campaign in May, persisting consistently thereafter. Significantly higher *V. harveyi* concentrations was observed in August compared to other months (*p* < 0.05). Of note, the *V. harveyi* abundance in the bottom layer ranged from 0.52 to 3.36 log(GE·mL^−1^) (Figure 1), with a distinct peak in August, when the temperature of the water tank was at its highest. Moreover, the abundances in the middle and top layers exhibited similar profiles through the months, ranging from 0.60 to 2.43 log(GE·mL^−1^) and from 0.48 to 2.89 log(GE·mL^−1^), respectively.

Many samples were found to be below the qPCR detection threshold for the concrete samples. The estimated concentration of *V. harveyi* in the biofilm samples did not exhibit a significant difference over time and through the water column. *Vibrio harveyi* started to be detected in June and ceased by the end of October for the top and middle tank samples. The bottom samples were detected until the end of the sampling campaign. The detected abundances in the bottom, middle and top layers of the tank ranged from 2.88 to 4.51 log(GE·cm^−2^), 3.02 to 4.60 log(GE·cm^−2^), and 2.98 to 4.93 log(GE·cm^−2^), respectively (Figure 1). A rapid increase in *V. harveyi* abundance was observed during high-temperature periods, particularly from August to September, resulting in two peaks during these months associated with fish mortality in the tank (Table 1). Statistical analysis revealed significant variations in the *V. harveyi* concentrations for all the layers, with August and September forming a cluster characterised by concentrations significantly higher than those observed for the rest of the months (*p* < 0.05). Moreover, during these mortality episodes, oxytetracycline was used and a resulting decrease in *V. harveyi* abundance was observed, enabling the concentration maintenance to remain below 3.97 log(GE·cm^−2^) for the concrete samples and 1.90 log(GE·mL^−1^) for the water samples. The abundance of *V. harveyi* gradually rose as the antibiotic’s predicted effect started to fade.

### 3.3. Influence of Environmental Factors

**Figure 2:**
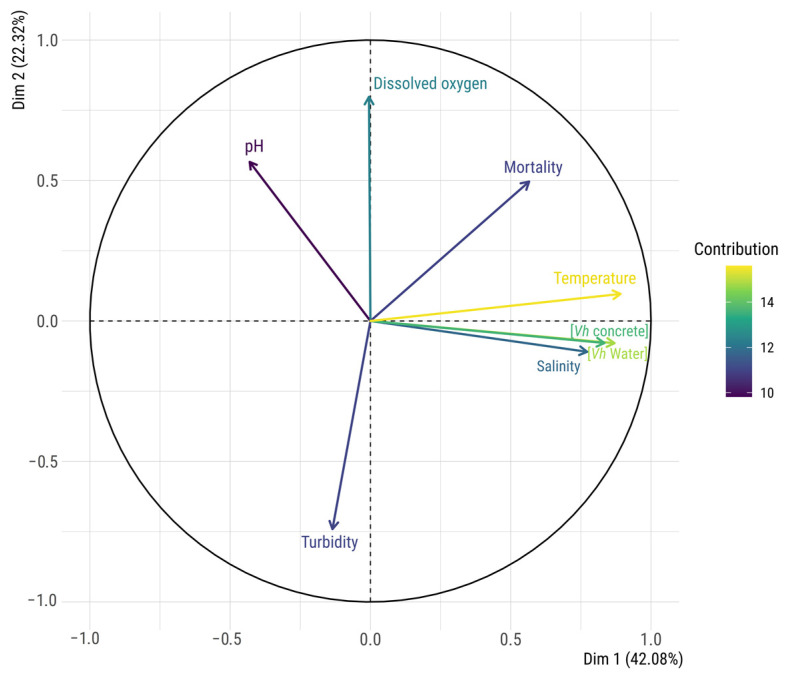
Circle of correlation of the PCA for parameters, including *V. harveyi* in water ([*Vh* water]), *V. harveyi* in biofilms ([*Vh* concrete]), dissolved oxygen, temperature, salinity, turbidity, pH, and fish mortality (Mortality). The contribution of each variable indicates its importance in explaining the overall variance in the dataset.

**Figure 3:**
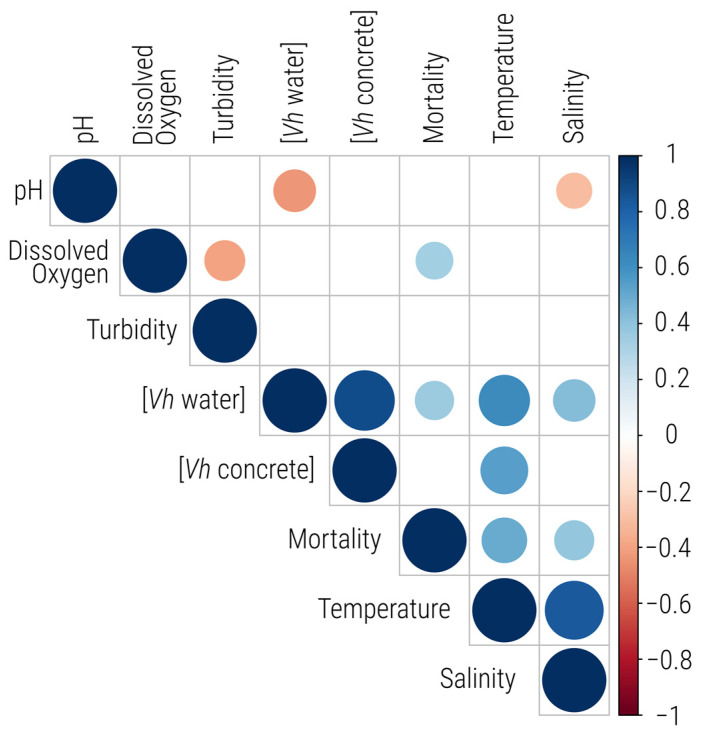
Correlation matrix of tank environmental parameters, including *V. harveyi* concentrations in water ([*Vh* Water]), *V. harveyi* in biofilms ([*Vh* Concrete]), dissolved oxygen, temperature, salinity, turbidity, pH, and fish mortality (Mortality). The size of the circles corresponds to the strength of the correlation, with larger circles indicating stronger correlations and small ones indicating weak correlations. Only statistically significant correlations (*p* < 0.05) between the parameters are shown.

PCA (Figure 2) was combined with a correlation matrix (Figure 3) to reveal the interactions of the parameters measured in the aquaculture tank. Both dimensions of the PCA, Dim.1 and Dim.2, captured 64.40% of the data variance, explaining 42.08% and 22.32% of the total variance, respectively. Temperature, *V. harveyi* abundance in water ([*Vh* water]), and *V. harveyi* in biofilm form ([*Vh* concrete]) emerged as the primary drivers shaping the Dim.1 variance landscape, while Dim.2 was predominantly influenced by DO and turbidity. Strong positive correlations (0.88, *p* < 0.001) were found between *V. harveyi* abundance in water and its abundance in biofilm within the tank, as highlighted in the correlation matrix, indicating that the biofilm results closely align with those observed in the water. A strong positive correlation was observed in the PCA between temperature and both *V. harveyi* abundance in water (0.891, *p* < 0.001) and in biofilm (0.834, *p* < 0.001), but also, to a lesser extent, salinity (0.772, *p* < 0.001). Further confirming and extending these findings, the correlation matrix highlighted significant positive correlations, such as temperature with salinity (0.84, *p* < 0.001), *V. harveyi* concentration in water (0.63, *p* < 0.001), concrete (0.55, *p* < 0.001), and fish mortality (0.50, *p* < 0.001). In contrast, pH exhibited a negative correlation with *V. harveyi* abundance in water (−0.43, *p* < 0.001), indicating an inverse relationship. On the other hand, Dim.2 revealed significant correlations with DO (0.798, *p* < 0.001), pH (0.566, *p* < 0.001), fish mortality (0.496, *p* < 0.001), and turbidity (−0.741, *p* < 0.001). Additionally, the correlation matrix revealed strong negative correlations between DO and turbidity (−0.40, *p* = 0.0054), and positive correlation with fish mortality (0.34, *p* = 0.0196).

## 4. Discussion

### 4.1. Temporal and Spatial Study of V. harveyi in Aquaculture

Our investigation examined the temporal and spatial distribution of *V. harveyi* throughout a sampling campaign spanning May to November 2022 on a farm in northern France. This farm has experienced recurring vibriosis episodes attributed to *V. harveyi*. A previous study conducted in a “small tank” (75 m^3^, 1.3 m deep) revealed the consistent presence and persistence of *V. harveyi* in both planktonic form and biofilms throughout the year [14]. This previous study was restricted by the absence of vibriosis outbreaks during the campaign, but also by the small tank size, which prevented in-depth exploration of the variations in environmental conditions and their impact on *V. harveyi*. To better understand the dynamics of *V. harveyi* throughout the water column, we implemented a sampling campaign in a 4-metre-deep tank at three different depths. We systematically monitored the environmental parameters to find potential explanations for the observed variations. Our study sets itself apart by adopting a comprehensive sampling approach over an extended time frame that included the periods before, during, and after two episodes of vibriosis. Additionally, during our sampling campaign, the two vibriosis outbreaks forced the farmers to use antibiotics. Consequently, this study offers insights into the antibiotic effects on *V. harveyi*, considering both the planktonic and biofilm forms. This campaign also sheds light on the omnipresence of *V. harveyi* within the aquaculture tank. From the very onset, brought in with the initial filling of the tank with water, *V. harveyi* demonstrated its adaptability, swiftly establishing its presence in the tank environment. *Vibrio harveyi* was present and quantifiable during the whole sampling campaign in planktonic form in the water, and during the warmer months in biofilms. Its abundance in biofilms potentially increased due to persistence once established on the tank wall, a finding in line with the previous sampling campaign on the same aquaculture farm in 2018 [14]. A distinctive characteristic of *Vibrio*, including *V. harveyi*, is their ability to adapt to changing conditions. This adaptability is underscored by their capacity to transition from free-swimming cells to “swarmer cells” within biofilms [25,26]. Furthermore, during the warmer months, it is known that the abundance of other coexisting bacterial families within biofilms, such as *Rhodobacteraceae*, decreases, providing an environment that enables the *Vibrionaceae* to thrive in the microbial community structure [15,27].

### 4.2. Abundance of V. harveyi in the Tank

In comparison to Mougin et al. [14], our study observed higher concentrations of *V. harveyi* in our water tank. They actually found a maximum concentration of 9.51 × 10^1^ GE.mL^−1^ in rearing tanks, while a maximum concentration of 2.54 × 10^3^ GE.ml^−1^ was found in our water tank. This contrast could be due to differences in the environmental conditions, but also due to the different tank sizes and fish. In our study, which aimed to investigate the water column, all the measured abiotic parameters in the water column exhibited clear stability, a characteristic likely shaped by the conditions of fish farming. These conditions, which ensure efficient water circulation, additionally play a role in the homogenising tank parameters overall. The concentrations of *V. harveyi* reflected the same stability throughout the water column. Similar observations regarding *Vibrio* spp. concentrations in the water column were found in a natural oyster farming site [28]. The only significant difference observed by Cruz et al. [28] appeared for some oysters collected close to the seafloor, where the sediment accumulation was high. Even though our tank water appeared to be homogeneous, organic matter accumulated on the floor of the tank. This phenomenon is caused by fewer fish activities at the bottom, resulting in less associated movement. It can also be attributed to the sedimentation and accumulation of nutrient-rich sediments consisting of food scraps and fish faeces. Moreover, sediments are known to serve as a source of nutrients for *V. harveyi*, which not only sediment themselves in the tank but are also able to absorb dissolved organic matter [29]. Sediments also provide an additional surface for colonisation, protecting the bacteria from biotic and abiotic stresses and enabling their persistence in the tank [30,31]. This hypothesis regarding the sediment dynamics and *V. harveyi* colonisation could be tested by conducting further sampling after emptying the tank, which would provide insights into the role of sediments in the *V. harveyi* dynamics in aquaculture settings.

### 4.3. Environmental Factors Influencing V. harveyi Distribution

Even though the *V. harveyi* concentrations were spatially homogeneous throughout the water column, temporal variations were observed throughout the 7-month sampling campaign. The relationships between the bacterial concentrations and measured environmental parameters were investigated through PCA (Figure 2) and a correlation matrix (Figure 3). Interestingly, temperature emerged as the foremost factor influencing the abundance of *V. harveyi*, as the abundance was consistently noted to be more favourable as the temperature rose above 20 °C. Given that *V. harveyi* was introduced into the tank water from the sea, its persistence within the tank is facilitated by favourable tank conditions, enabling it to form biofilms on the tank walls and promoting its growth. Additionally, extensive studies have demonstrated the crucial influence of temperature on the presence of *V. harveyi* in natural aquatic systems, displaying a clear seasonal cycle, particularly during the summer when the temperature exceeds 25 °C [32,33]. This seasonality was also observed in our study, as *V. harveyi* exhibited higher abundance during the warmest months, when the temperature reached up to 22 °C. However, it is important to note that vibriosis does not necessarily occur when the temperature reaches 22 °C, suggesting that additional factors may influence its occurrence. Additionally, the correlation matrix (Figure 3) suggests that salinity and pH, which are predicted to change with the overall ocean temperature [34], also positively affected the presence of *V. harveyi* in the aquaculture tank. These findings are consistent with prior research on the effects of environmental parameters on *Vibrio* species in natural and aquaculture settings, where a positive correlation between salinity and temperature with the frequency of detection of *Vibrio* species has been reported [35,36]. The DO concentration is reported to have a negative correlation with the temperature, given that increased water temperatures lead to decreased DO levels [37]. However, in our case, the DO levels were continuously managed by the farmers, which explains the absence of any observable effect on the abundance of *V. harveyi.* A second negative correlation was observed with the pH values. Although *V. harveyi* is known to tolerate wide ranges of pH levels (i.e., 5.5 to 9.0), the correlation matrix in our study shows that pH is negatively correlated with both *V. harveyi* abundance in water and salinity [38]. This parameter cannot be fully controlled in aquaculture, adding another challenge to vibriosis management in tanks. In addition, global warming and ocean acidification may alter the microbial balance, favouring the presence of *Vibrio* while diminishing populations of other bacterial genera [12]. All these observations highlight the complexity of identifying the multiparameter ideal *Vibrio* environment, which varies based on geographic location and environmental conditions [39].

### 4.4. Challenges and Considerations in Managing Vibriosis Outbreaks

The repeated occurrences and unpredictability of vibriosis outbreaks associated with *V. harveyi* present a significant challenge to the seabass aquaculture sector, as highlighted by Vendramin et al. [11] and Firmino et al. [40]. This issue becomes particularly problematic in open-system aquaculture, since the prevalence of this pathogen in fish closely aligns with its abundance in the surrounding environment [41], but also because the water quality cannot be fully controlled. Moreover, in the context of climate change, there has been a temperature increase of about 0.88 °C in the sea and ocean surface temperatures over the last century [34,42]. This temperature rise is known to affect the distribution and abundance of marine organisms [43], including *Vibrio*, which has seen its abundance increase sharply in the North Sea over the period 1958–2011 [31]. According to the Intergovernmental Panel on Climate Change, the ocean is projected to continue warming, leading to long-term impacts on ocean ecosystems and human societies [43]. Open aquaculture systems may therefore need to address an increase in vibriosis episodes. This heightened risk could require increased antibiotic use, posing risks to fish health, environmental integrity, and consumer safety. In response to these challenges, it is imperative to develop specific and effective solutions. Antibiotics, as suggested by our findings, fall short as a sustainable solution, since despite their use by the farmers, the concentrations of *V. harveyi* in the water and biofilms quickly rebounded. Within a three-month period, the farmers had to resort to antibiotics twice to avoid massive fish losses (Figure 2). Given the escalating importance of addressing the *Vibrio* challenge in response to global warming, it is crucial to adopt a comprehensive approach to understand the virulence mechanisms used by *V. harveyi.* As an opportunistic pathogen, *V. harveyi* thrives in warmer temperatures, requiring proactive measures in response to changing climate conditions that could now favour its growth in previously cooler waters. Addressing the core issue involves reducing bacterial virulence through antivirulence therapies [44,45]. Environmental effects impact not only the behaviour of the bacterium but also its area of distribution, which is bound to evolve. For some geographic areas, including the one in the present study and more generally temperate waters, such changes may pose challenges regarding *Vibrio* expansion [39]. Moreover, they may even compromise host immunity, thereby enhancing the bacterium’s potential to increase mortality. Understanding these multifaceted interactions becomes a keystone in formulating nuanced and effective strategies aimed at mitigating the impact of potential vibriosis outbreaks.

## 5. Conclusions

In conclusion, this study highlighted the challenges of managing *V. harveyi* outbreaks in aquaculture systems. The persistence of *V. harveyi* poses major challenges, particularly during warmer months. The high adaptability of *Vibrio*, and particularly *V. harveyi*, implies its considerable permissiveness, rendering the task of attempting to control the entire environment to prevent vibriosis highly challenging, especially in the context of global warming and ocean acidification [12]. Additionally, significant correlations were found between the *V. harveyi* concentrations and various environmental parameters, highlighting the complex dynamics influencing its distribution. While temperature emerged as a primary influencer, the absence of vibriosis outbreaks every summer suggests the influence of additional parameters such as salinity and pH. Furthermore, in the context of the escalating *Vibrio* abundance linked to rising global ocean temperatures, concerns arise regarding the heightened pathogenic potential of certain *Vibrio* strains, potentially leading to an increased incidence of diseases in the marine environment. This poses a real challenge for open aquaculture systems, which are specifically impacted by these temperature increases as they are unable to regulate the water temperature during the hottest months. This study aimed to conduct a diagnostic assessment of the tank environment and investigate the spatio-temporal dynamics of *V. harveyi* to gain a deeper understanding of the pathogen–environment interactions that could lead to vibriosis outbreaks. In navigating these intricate ecological challenges, the integration of a “One Health” framework becomes imperative, emphasising the interconnectedness of animal, environmental, and human health [46,47]. Understanding the virulence mechanisms of *V. harveyi* is crucial for developing effective countermeasures and adaptive strategies that align with changing ecological systems. This emphasises the need to explore its virulence genes and their responses to environmental factors like temperature, which are expected to evolve in the future.

## Figures and Tables

**Table 1:** Mean values ± standard deviations of the parameters recorded from May to November 2022 in the aquaculture facilities, categorised by the top, middle (mid), and bottom (bot) layers of the rearing tank. The ‘*n*’ signifies the number of sampling times averaged per month, knowing that a higher sampling frequency was used when water temperature exceeded 19 °C. For each sampling time and each layer, the average was calculated from 10 probe measurements.

		Sampling Month						
		May (*n* = 2)	June (*n* = 2)	July (*n* = 3)	August (*n* = 4)	September (*n* = 2)	October (*n* = 2)	November (*n* = 1)
Temperature (°C)	Top	18.29 ± 0.77	19.06 ± 0.30	20.89 ± 0.28	22.06 ± 0.58	20.69 ± 0.03	17.82 ± 0.36	16.71 ± 0
	Mid	18.29 ± 0.74	19.06 ± 0.30	20.89 ± 0.28	22.05 ± 0.57	20.71 ± 0.05	17.82 ± 0.36	16.70 ± 0
	Bot	18.29 ± 0.74	19.06 ± 0.30	20.89 ± 0.28	22.06 ± 0.57	20.71 ± 0.04	17.82 ± 0.36	16.70 ± 0
pH	Top	8.07 ± 0.71	7.53 ± 0.16	7.37 ± 0.22	7.29 ± 0.04	7.39 ± 0.06	7.17 ± 0.19	7.06 ± 0
	Mid	8.06 ± 0.70	7.55 ± 0.06	7.31 ± 0.24	7.23 ± 0.07	7.33 ± 0.03	7.12 ± 0.17	7.03 ± 0
	Bot	7.95 ± 0.72	7.51 ± 0.02	7.25 ± 0.23	7.18 ± 0.07	7.25 ± 0.08	7.02 ± 0.16	6.87 ± 0
Salinity (psu)	Top	33.83 ± 0.68	34.75 ± 0.16	35.14 ± 0.16	35.07 ± 0.16	34.97 ± 0.11	34.09 ± 0.05	33.08 ± 0
	Mid	33.40 ± 1.34	34.74 ± 0.18	35.17 ± 0.18	35.16 ± 0.07	34.91 ± 0.05	34.00 ± 0.18	31.99 ± 0
	Bot	33.45 ± 1.34	34.63 ± 0.27	35.12 ± 0.15	35.18 ± 0.10	34.83 ± 0.20	33.81 ± 0.30	32.67 ± 0
Dissolved oxygen (mg.L^−1^)	Top	5.27 ± 0.15	4.13 ± 0.04	3.20 ± 0.01	4.70 ± 0.21	5.46 ± 0.02	3.47 ± 0.06	6.47 ± 0.15
	Mid	5.45 ± 0.03	5.19 ± 0.02	3.39 ± 0.01	3.63 ± 0.02	5.83 ± 0.09	3.46 ± 0.04	3.64 ± 0.26
	Bot	5.74 ± 0.03	5.49 ± 0.03	3.44 ± 0.02	4.04 ± 0.03	7.30 ± 0.43	3.82 ± 0.05	3.65 ± 0.11
Turbidity (FNU)	Top	4.51 ± 1.02	5.02 ± 0.07	4.90 ± 1.44	2.84 ± 0.82	6.89 ± 0.52	7.15 ± 4.95	3.30 ± 0
	Mid	4.26 ± 1.32	3.40 ± 1.45	4.17 ± 0.75	3.86 ± 0.83	4.64 ± 1.65	5.56 ± 2.36	6.67 ± 0
	Bot	3.99 ± 0.47	3.99 ± 0.77	6.41 ± 2.73	3.83 ± 0.80	6.39 ± 3.13	5.35 ± 1.77	5.29 ± 0
Fish Mortality (%)		1.26	0.98	1.31	4.57	0.90	0.06	0.01

## Data Availability

The original contributions presented in the study are included in the article/Supplementary Material. Further inquiries can be directed to the corresponding author.

## Supplementary Materials

The following supporting information can be downloaded at https://www.mdpi.com/article/10.3390/microorganisms12061104/s1, Table S1: Sampling dates; Table S2: Details of the samples and concentrations of the *V. harveyi* genome copy equivalents (GE)·mL^−1^ for water samples and log GE·cm^−2^ for concrete samples, as obtained during the sampling campaign.
