# Supplementary material for: Temporal and Spatial Dynamics of Vibrio harveyi: An Environmental Parameter Correlation Investigation in a 4-Metre-Deep Dicentrarchus labrax Aquaculture Tank
Source: Microorganisms. 2024 May 29;12(6):1104. doi: 10.3390/microorganisms12061104 (PMC11205727; doi:10.3390/microorganisms12061104)
Supplement: Supplementary file 1 [file microorganisms-12-01104-s001.zip › microorganisms-2993375-supplementary.pdf]

Table S1. Sampling dates. Samplings in heatwaves are highlighted in red. Heatwaves are characterised as periods when the seawater temperature exceeded 19 °C for two consecutive nights, requiring scheduled sampling within the following 4–5 days.

**Sampling number**	**Month**	**Sampling date**
Sampling 0	May	2022-05-11
Sampling 1	May	2022-05-18
Sampling 2	June	2022-06-08
Sampling 3	June	2022-06-23
Sampling 4	July	2022-07-13
**Sampling 5**	**July**	**2022-07-20**
**Sampling 6**	**July**	**2022-07-27**
**Sampling 7**	**August**	**2022-08-03**
**Sampling 8**	**August**	**2022-08-10**
**Sampling 9**	**August**	**2022-08-17**
**Sampling 10**	**August**	**2022-08-24**
**Sampling 11**	**September**	**2022-09-01**
Sampling 12	September	2022-09-15
Sampling 13	October	2022-10-05
Sampling 14	October	2022-10-26
Sampling 15	November	2022-11-15

Table S2. Details of samples and concentrations of V. harveyi genome copy equivalents (GE).mL $^{-1}$ for water samples and log GE.cm $^{-2}$ for concrete samples, obtained during the sampling campaign. C Dry: non-immersed concrete specimens.

Sample	Date	Month	Medium	Depth	GE.mL $^{-1}$ qPCR replicate 1	GE.mL $^{-1}$ qPCR replicate 2
W0B1	2022-05-11	May	Water	Bottom	2.97E+01	2.45E+01
W0B2	2022-05-11	May	Water	Bottom	4.38E+00	3.40E+00
W0B3	2022-05-11	May	Water	Bottom	1.00E+01	1.07E+01
W0B4	2022-05-11	May	Water	Bottom	1.82E+01	1.55E+01
W0B5	2022-05-11	May	Water	Bottom	1.55E+01	1.24E+01
W0M1	2022-05-11	May	Water	Middle	7.42E+00	5.99E+00
W0M2	2022-05-11	May	Water	Middle	4.05E+00	6.15E+00
W0M3	2022-05-11	May	Water	Middle	5.46E+00	7.42E+00
W0M4	2022-05-11	May	Water	Middle	1.21E+01	9.62E+00
W0M5	2022-05-11	May	Water	Middle	6.11E+00	4.07E+00
W0T1	2022-05-11	May	Water	Top	2.40E+01	2.04E+01
W0T2	2022-05-11	May	Water	Top	2.94E+00	3.38E+00
W0T3	2022-05-11	May	Water	Top	5.46E+00	5.32E+00
W0T4	2022-05-11	May	Water	Top	1.30E+01	6.67E+00
W0T5	2022-05-11	May	Water	Top	6.45E+00	3.71E+00
W1B1	2022-05-18	May	Water	Bottom	1.16E+02	9.15E+01
W1B2	2022-05-18	May	Water	Bottom	1.09E+02	9.78E+01
W1B3	2022-05-18	May	Water	Bottom	2.10E+01	2.41E+01
W1B4	2022-05-18	May	Water	Bottom	7.25E+01	8.56E+01
W1B5	2022-05-18	May	Water	Bottom	2.70E+01	2.26E+01
W1M1	2022-05-18	May	Water	Middle	2.70E+01	1.85E+01
W1M2	2022-05-18	May	Water	Middle	3.65E+01	2.48E+01
W1M3	2022-05-18	May	Water	Middle	1.84E+01	2.35E+01
W1M4	2022-05-18	May	Water	Middle	2.03E+01	1.68E+01
W1M5	2022-05-18	May	Water	Middle	1.36E+01	1.02E+01
W1T1	2022-05-18	May	Water	Top	5.59E+01	3.65E+01
W1T2	2022-05-18	May	Water	Top	4.83E+01	3.51E+01
W1T3	2022-05-18	May	Water	Top	1.90E+01	1.05E+01
W1T4	2022-05-18	May	Water	Top	1.45E+01	1.03E+01
W1T5	2022-05-18	May	Water	Top	2.06E+01	1.54E+01
W2B1	2022-06-08	June	Water	Bottom	8.97E+01	6.96E+01
W2B2	2022-06-08	June	Water	Bottom	5.82E+01	5.30E+01
W2B3	2022-06-08	June	Water	Bottom	1.02E+02	8.45E+01
W2B4	2022-06-08	June	Water	Bottom	8.73E+01	5.89E+01
W2B5	2022-06-08	June	Water	Bottom	2.02E+01	1.42E+01
W2M1	2022-06-08	June	Water	Middle	6.36E+00	2.47E+00
W2M2	2022-06-08	June	Water	Middle	2.45E+01	1.56E+01
W2M3	2022-06-08	June	Water	Middle	1.42E+01	7.27E+00
W2M4	2022-06-08	June	Water	Middle	4.57E+01	3.84E+01
W2M5	2022-06-08	June	Water	Middle	1.04E+02	9.58E+01

W2T1	2022-06-08	June	Water	Top	4.18E+01	2.91E+01
W2T2	2022-06-08	June	Water	Top	2.97E+01	2.51E+01
W2T3	2022-06-08	June	Water	Top	1.74E+01	1.73E+01
W2T4	2022-06-08	June	Water	Top	2.54E+01	2.84E+01
W2T5	2022-06-08	June	Water	Top	1.54E+01	1.64E+01
W3B1	2022-06-23	June	Water	Bottom	9.04E+01	9.28E+01
W3B2	2022-06-23	June	Water	Bottom	1.65E+01	1.24E+01
W3B3	2022-06-23	June	Water	Bottom	4.17E+00	2.67E+00
W3B4	2022-06-23	June	Water	Bottom	6.76E+00	7.99E+00
W3B5	2022-06-23	June	Water	Bottom	1.97E+01	2.54E+01
W3M1	2022-06-23	June	Water	Middle	4.50E+00	8.30E+00
W3M2	2022-06-23	June	Water	Middle	5.02E+00	5.50E+00
W3M3	2022-06-23	June	Water	Middle	7.16E+00	6.21E+00
W3M4	2022-06-23	June	Water	Middle	7.83E+00	8.80E+00
W3M5	2022-06-23	June	Water	Middle	3.76E+00	6.09E+00
W3T1	2022-06-23	June	Water	Top	4.39E+00	5.94E+00
W3T2	2022-06-23	June	Water	Top	8.68E+00	9.50E+00
W3T3	2022-06-23	June	Water	Top	5.83E+00	5.02E+00
W3T4	2022-06-23	June	Water	Top	4.14E+00	4.56E+00
W3T5	2022-06-23	June	Water	Top	7.83E+00	7.54E+00
W4B1	2022-07-13	July	Water	Bottom	4.54E+01	4.98E+01
W4B2	2022-07-13	July	Water	Bottom	1.44E+01	1.34E+01
W4B3	2022-07-13	July	Water	Bottom	2.53E+01	2.33E+01
W4B4	2022-07-13	July	Water	Bottom	1.70E+01	2.11E+01
W4B5	2022-07-13	July	Water	Bottom	2.88E+01	2.97E+01
W4M1	2022-07-13	July	Water	Middle	2.41E+02	2.24E+02
W4M2	2022-07-13	July	Water	Middle	7.39E+00	8.09E+00
W4M3	2022-07-13	July	Water	Middle	2.27E+01	1.51E+01
W4M4	2022-07-13	July	Water	Middle	1.97E+01	1.42E+01
W4M5	2022-07-13	July	Water	Middle	1.52E+01	1.80E+01
W4T1	2022-07-13	July	Water	Top	9.08E+00	5.19E+00
W4T2	2022-07-13	July	Water	Top	1.19E+01	1.15E+01
W4T3	2022-07-13	July	Water	Top	1.09E+01	8.80E+00
W4T4	2022-07-13	July	Water	Top	5.75E+00	7.73E+00
W4T5	2022-07-13	July	Water	Top	1.27E+01	1.11E+01
W5B1	2022-07-20	July	Water	Bottom	8.04E+00	5.26E+00
W5B2	2022-07-20	July	Water	Bottom	4.33E+01	3.60E+01
W5B3	2022-07-20	July	Water	Bottom	2.38E+01	1.67E+01
W5B4	2022-07-20	July	Water	Bottom	1.45E+01	6.81E+00
W5B5	2022-07-20	July	Water	Bottom	1.70E+01	9.87E+00
W5M1	2022-07-20	July	Water	Middle	1.73E+01	1.67E+01
W5M2	2022-07-20	July	Water	Middle	1.45E+01	1.51E+01
W5M3	2022-07-20	July	Water	Middle	1.36E+01	1.37E+01
W5M4	2022-07-20	July	Water	Middle	3.67E+01	2.63E+01
W5M5	2022-07-20	July	Water	Middle	1.75E+01	1.88E+01
W5T1	2022-07-20	July	Water	Top	1.79E+01	1.89E+01
W5T2	2022-07-20	July	Water	Top	7.72E+00	8.76E+00

W5T3	2022-07-20	July	Water	Top	2.45E+01	1.88E+01
W5T4	2022-07-20	July	Water	Top	1.66E+01	1.54E+01
W5T5	2022-07-20	July	Water	Top	3.13E+00	3.95E+00
W6B1	2022-07-27	July	Water	Bottom	7.56E+01	7.03E+01
W6B2	2022-07-27	July	Water	Bottom	6.41E+01	5.65E+01
W6B3	2022-07-27	July	Water	Bottom	1.38E+02	8.03E+01
W6B4	2022-07-27	July	Water	Bottom	4.14E+01	3.57E+01
W6B5	2022-07-27	July	Water	Bottom	1.93E+02	1.18E+02
W6M1	2022-07-27	July	Water	Middle	1.77E+01	1.70E+01
W6M2	2022-07-27	July	Water	Middle	2.16E+01	1.78E+01
W6M3	2022-07-27	July	Water	Middle	1.84E+01	2.45E+01
W6M4	2022-07-27	July	Water	Middle	3.15E+01	2.55E+01
W6M5	2022-07-27	July	Water	Middle	3.46E+01	2.67E+01
W6T1	2022-07-27	July	Water	Top	7.98E+01	7.66E+01
W6T2	2022-07-27	July	Water	Top	1.56E+01	1.71E+01
W6T3	2022-07-27	July	Water	Top	3.28E+01	4.11E+01
W6T4	2022-07-27	July	Water	Top	2.42E+01	1.75E+01
W6T5	2022-07-27	July	Water	Top	2.93E+01	3.41E+01
W7B1	2022-08-03	August	Water	Bottom	2.46E+01	2.14E+01
W7B2	2022-08-03	August	Water	Bottom	5.43E+01	3.62E+01
W7B3	2022-08-03	August	Water	Bottom	1.42E+01	1.14E+01
W7B4	2022-08-03	August	Water	Bottom	6.24E+01	6.24E+01
W7B5	2022-08-03	August	Water	Bottom	7.98E+01	4.95E+01
W7M1	2022-08-03	August	Water	Middle	2.91E+01	2.57E+01
W7M2	2022-08-03	August	Water	Middle	4.16E+01	3.41E+01
W7M3	2022-08-03	August	Water	Middle	8.30E+01	8.35E+01
W7M4	2022-08-03	August	Water	Middle	2.48E+01	2.19E+01
W7M5	2022-08-03	August	Water	Middle	2.68E+01	3.16E+01
W7T1	2022-08-03	August	Water	Top	4.77E+01	3.79E+01
W7T2	2022-08-03	August	Water	Top	1.09E+01	7.92E+00
W7T3	2022-08-03	August	Water	Top	3.72E+01	3.39E+01
W7T4	2022-08-03	August	Water	Top	3.35E+01	2.94E+01
W7T5	2022-08-03	August	Water	Top	2.81E+01	1.79E+01
W8B1	2022-08-10	August	Water	Bottom	1.17E+02	1.03E+02
W8B2	2022-08-10	August	Water	Bottom	2.39E+02	3.58E+02
W8B3	2022-08-10	August	Water	Bottom	5.72E+01	4.99E+01
W8B4	2022-08-10	August	Water	Bottom	1.57E+02	1.16E+02
W8B5	2022-08-10	August	Water	Bottom	6.15E+01	6.56E+01
W8M1	2022-08-10	August	Water	Middle	3.37E+01	3.22E+01
W8M2	2022-08-10	August	Water	Middle	3.94E+01	3.14E+01
W8M3	2022-08-10	August	Water	Middle	5.43E+01	5.76E+01
W8M4	2022-08-10	August	Water	Middle	4.96E+01	4.61E+01
W8M5	2022-08-10	August	Water	Middle	8.63E+01	9.21E+01
W8T1	2022-08-10	August	Water	Top	5.68E+01	4.64E+01
W8T2	2022-08-10	August	Water	Top	3.84E+01	4.44E+01
W8T3	2022-08-10	August	Water	Top	6.56E+01	5.26E+01
W8T4	2022-08-10	August	Water	Top	8.36E+02	7.20E+02

W8T5	2022-08-10	August	Water	Top	6.43E+01	5.36E+01
W9B1	2022-08-17	August	Water	Bottom	7.67E+01	5.36E+01
W9B2	2022-08-17	August	Water	Bottom	2.54E+03	2.23E+03
W9B3	2022-08-17	August	Water	Bottom	1.76E+02	2.03E+02
W9B4	2022-08-17	August	Water	Bottom	7.73E+02	5.51E+02
W9B5	2022-08-17	August	Water	Bottom	8.77E+02	7.51E+02
W9M1	2022-08-17	August	Water	Middle	7.67E+01	5.65E+01
W9M2	2022-08-17	August	Water	Middle	8.25E+01	5.32E+01
W9M3	2022-08-17	August	Water	Middle	2.70E+02	2.72E+02
W9M4	2022-08-17	August	Water	Middle	5.54E+01	5.99E+01
W9M5	2022-08-17	August	Water	Middle	9.09E+01	9.21E+01
W9T1	2022-08-17	August	Water	Top	1.51E+02	1.20E+02
W9T2	2022-08-17	August	Water	Top	7.33E+01	5.57E+01
W9T3	2022-08-17	August	Water	Top	1.47E+02	1.22E+02
W9T4	2022-08-17	August	Water	Top	1.79E+02	1.54E+02
W9T5	2022-08-17	August	Water	Top	8.09E+01	8.63E+01
W10B1	2022-08-24	August	Water	Bottom	2.48E+02	2.08E+02
W10B2	2022-08-24	August	Water	Bottom	1.18E+02	1.48E+02
W10B3	2022-08-24	August	Water	Bottom	2.25E+02	1.61E+02
W10B4	2022-08-24	August	Water	Bottom	1.61E+02	1.30E+02
W10B5	2022-08-24	August	Water	Bottom	1.81E+02	1.19E+02
W10M1	2022-08-24	August	Water	Middle	1.10E+02	8.07E+01
W10M2	2022-08-24	August	Water	Middle	1.62E+02	1.48E+02
W10M3	2022-08-24	August	Water	Middle	9.35E+01	9.35E+01
W10M4	2022-08-24	August	Water	Middle	5.78E+01	5.25E+01
W10M5	2022-08-24	August	Water	Middle	9.91E+01	8.23E+01
W10T1	2022-08-24	August	Water	Top	1.98E+02	1.82E+02
W10T2	2022-08-24	August	Water	Top	1.31E+02	1.10E+02
W10T3	2022-08-24	August	Water	Top	7.06E+01	5.12E+01
W10T4	2022-08-24	August	Water	Top	1.26E+02	1.36E+02
W10T5	2022-08-24	August	Water	Top	2.68E+02	1.59E+02
W11B1	2022-09-01	September	Water	Bottom	8.61E+01	7.47E+01
W11B2	2022-09-01	September	Water	Bottom	3.96E+01	3.15E+01
W11B3	2022-09-01	September	Water	Bottom	3.19E+01	3.69E+01
W11B4	2022-09-01	September	Water	Bottom	5.97E+01	6.41E+01
W11B5	2022-09-01	September	Water	Bottom	1.81E+01	1.47E+01
W11M1	2022-09-01	September	Water	Middle	5.02E+01	3.46E+01
W11M2	2022-09-01	September	Water	Middle	3.89E+01	3.51E+01
W11M3	2022-09-01	September	Water	Middle	5.15E+01	4.34E+01
W11M4	2022-09-01	September	Water	Middle	5.57E+01	5.12E+01
W11M5	2022-09-01	September	Water	Middle	4.25E+01	4.23E+01
W11T1	2022-09-01	September	Water	Top	6.79E+01	4.28E+01
W11T2	2022-09-01	September	Water	Top	2.95E+01	2.11E+01
W11T3	2022-09-01	September	Water	Top	2.17E+01	2.11E+01
W11T4	2022-09-01	September	Water	Top	1.92E+01	2.28E+01
W11T5	2022-09-01	September	Water	Top	1.63E+01	1.47E+01
W12B1	2022-09-15	September	Water	Bottom	9.54E+01	6.37E+01

W12B2	2022-09-15	September	Water	Bottom	2.70E+01	3.11E+01
W12B3	2022-09-15	September	Water	Bottom	1.22E+02	1.11E+02
W12B4	2022-09-15	September	Water	Bottom	1.08E+02	9.23E+01
W12B5	2022-09-15	September	Water	Bottom	5.19E+01	3.44E+01
W12M1	2022-09-15	September	Water	Middle	8.66E+01	7.87E+01
W12M2	2022-09-15	September	Water	Middle	9.78E+01	6.58E+01
W12M3	2022-09-15	September	Water	Middle	8.55E+01	7.43E+01
W12M4	2022-09-15	September	Water	Middle	8.44E+01	7.72E+01
W12M5	2022-09-15	September	Water	Middle	1.42E+02	1.19E+02
W12T1	2022-09-15	September	Water	Top	8.19E+01	7.02E+01
W12T2	2022-09-15	September	Water	Top	1.29E+02	1.14E+02
W12T3	2022-09-15	September	Water	Top	1.01E+02	8.74E+01
W12T4	2022-09-15	September	Water	Top	5.02E+01	3.98E+01
W12T5	2022-09-15	September	Water	Top	5.64E+01	5.02E+01
W13B1	2022-10-05	October	Water	Bottom	4.58E+01	4.06E+01
W13B2	2022-10-05	October	Water	Bottom	4.99E+01	3.30E+01
W13B3	2022-10-05	October	Water	Bottom	5.22E+01	4.22E+01
W13B4	2022-10-05	October	Water	Bottom	1.85E+01	2.10E+01
W13B5	2022-10-05	October	Water	Bottom	4.99E+01	4.99E+01
W13M1	2022-10-05	October	Water	Middle	2.20E+01	1.22E+01
W13M2	2022-10-05	October	Water	Middle	8.97E+00	9.38E+00
W13M3	2022-10-05	October	Water	Middle	1.31E+01	1.18E+01
W13M4	2022-10-05	October	Water	Middle	1.37E+01	1.24E+01
W13M5	2022-10-05	October	Water	Middle	1.24E+01	7.78E+00
W13T1	2022-10-05	October	Water	Top	2.30E+01	1.60E+01
W13T2	2022-10-05	October	Water	Top	1.36E+01	1.16E+01
W13T3	2022-10-05	October	Water	Top	1.01E+01	9.03E+00
W13T4	2022-10-05	October	Water	Top	1.57E+01	1.53E+01
W13T5	2022-10-05	October	Water	Top	1.82E+00	4.95E+00
W14B1	2022-10-26	October	Water	Bottom	3.07E+01	4.08E+01
W14B2	2022-10-26	October	Water	Bottom	1.31E+01	1.39E+01
W14B3	2022-10-26	October	Water	Bottom	1.57E+01	1.46E+01
W14B4	2022-10-26	October	Water	Bottom	1.35E+01	1.17E+01
W14B5	2022-10-26	October	Water	Bottom	6.21E+00	4.00E+00
W14M1	2022-10-26	October	Water	Middle	5.15E+00	3.63E+00
W14M2	2022-10-26	October	Water	Middle	2.37E+01	1.86E+01
W14M3	2022-10-26	October	Water	Middle	2.77E+01	2.96E+01
W14M4	2022-10-26	October	Water	Middle	1.98E+01	1.21E+01
W14M5	2022-10-26	October	Water	Middle	1.48E+01	9.03E+00
W14T1	2022-10-26	October	Water	Top	1.58E+01	2.23E+01
W14T2	2022-10-26	October	Water	Top	1.48E+01	1.07E+01
W14T3	2022-10-26	October	Water	Top	1.45E+01	1.30E+01
W14T4	2022-10-26	October	Water	Top	5.49E+01	4.74E+01
W14T5	2022-10-26	October	Water	Top	1.69E+01	1.46E+01
W15B1	2022-11-15	November	Water	Bottom	2.67E+01	3.63E+01
W15B2	2022-11-15	November	Water	Bottom	2.21E+01	2.26E+01
W15B3	2022-11-15	November	Water	Bottom	1.33E+01	1.28E+01

W15B4	2022-11-15	November	Water	Bottom	6.06E+01	5.14E+01
W15B5	2022-11-15	November	Water	Bottom	2.03E+02	1.99E+02
W15M1	2022-11-15	November	Water	Middle	6.49E+01	5.79E+01
W15M2	2022-11-15	November	Water	Middle	3.35E+01	2.46E+01
W15M3	2022-11-15	November	Water	Middle	1.70E+01	1.85E+01
W15M4	2022-11-15	November	Water	Middle	7.05E+01	4.98E+01
W15M5	2022-11-15	November	Water	Middle	3.75E+01	3.18E+01
W15T1	2022-11-15	November	Water	Top	2.22E+01	2.14E+01
W15T2	2022-11-15	November	Water	Top	3.75E+01	3.39E+01
W15T3	2022-11-15	November	Water	Top	3.61E+01	4.42E+01
W15T4	2022-11-15	November	Water	Top	4.26E+01	1.96E+01
W15T5	2022-11-15	November	Water	Top	6.25E+01	4.95E+01
**Sample**	**Date**	**Month**	**Medium**	**Depth**	**GE.mL $^{-1}$ qPCR replicate 1**	**GE.mL $^{-1}$ qPCR replicate 2**
C dry 1	2022-05-10	May	Concrete	/	0	0
C dry 2	2022-05-10	May	Concrete	/	0	0
C dry 3	2022-05-10	May	Concrete	/	0	0
C dry 4	2022-05-10	May	Concrete	/	0	0
C dry 5	2022-05-10	May	Concrete	/	0	0
C0B1	2022-05-11	May	Concrete	Bottom	0	0
C0B2	2022-05-11	May	Concrete	Bottom	0	0
C0B3	2022-05-11	May	Concrete	Bottom	0	0
C0B4	2022-05-11	May	Concrete	Bottom	0	0
C0B5	2022-05-11	May	Concrete	Bottom	0	0
C0M1	2022-05-11	May	Concrete	Middle	0	0
C0M2	2022-05-11	May	Concrete	Middle	0	0
C0M3	2022-05-11	May	Concrete	Middle	0	0
C0M4	2022-05-11	May	Concrete	Middle	0	0
C0M5	2022-05-11	May	Concrete	Middle	0	0
C0T1	2022-05-11	May	Concrete	Top	0	0
C0T2	2022-05-11	May	Concrete	Top	0	0
C0T3	2022-05-11	May	Concrete	Top	0	0
C0T4	2022-05-11	May	Concrete	Top	0	0
C0T5	2022-05-11	May	Concrete	Top	0	0
C1B1	2022-05-18	May	Concrete	Bottom	0	0
C1B2	2022-05-18	May	Concrete	Bottom	0	0
C1B3	2022-05-18	May	Concrete	Bottom	0	0
C1B4	2022-05-18	May	Concrete	Bottom	0	0
C1B5	2022-05-18	May	Concrete	Bottom	0	0
C1M1	2022-05-18	May	Concrete	Middle	0	0
C1M2	2022-05-18	May	Concrete	Middle	0	0
C1M3	2022-05-18	May	Concrete	Middle	0	0
C1M4	2022-05-18	May	Concrete	Middle	0	0
C1M5	2022-05-18	May	Concrete	Middle	0	0
C1T1	2022-05-18	May	Concrete	Top	0	0

C1T2	2022-05-18	May	Concrete	Top	0	0
C1T3	2022-05-18	May	Concrete	Top	0	0
C1T4	2022-05-18	May	Concrete	Top	0	0
C1T5	2022-05-18	May	Concrete	Top	0	0
C2B1	2022-06-08	June	Concrete	Bottom	0	0
C2B2	2022-06-08	June	Concrete	Bottom	0	0
C2B3	2022-06-08	June	Concrete	Bottom	0	0
C2B4	2022-06-08	June	Concrete	Bottom	0	0
C2B5	2022-06-08	June	Concrete	Bottom	0	0
C2M1	2022-06-08	June	Concrete	Middle	0	0
C2M2	2022-06-08	June	Concrete	Middle	0	0
C2M3	2022-06-08	June	Concrete	Middle	0	0
C2M4	2022-06-08	June	Concrete	Middle	0	0
C2M5	2022-06-08	June	Concrete	Middle	0	0
C2T1	2022-06-08	June	Concrete	Top	0	0
C2T2	2022-06-08	June	Concrete	Top	0	0
C2T3	2022-06-08	June	Concrete	Top	1.51E+03	8.43E+02
C2T4	2022-06-08	June	Concrete	Top	1.84E+03	1.26E+03
C2T5	2022-06-08	June	Concrete	Top	0	0
C3B1	2022-06-23	June	Concrete	Bottom	0	0
C3B2	2022-06-23	June	Concrete	Bottom	0	0
C3B3	2022-06-23	June	Concrete	Bottom	0	0
C3B4	2022-06-23	June	Concrete	Bottom	0	0
C3B5	2022-06-23	June	Concrete	Bottom	0	0
C3M1	2022-06-23	June	Concrete	Middle	0	0
C3M2	2022-06-23	June	Concrete	Middle	0	0
C3M3	2022-06-23	June	Concrete	Middle	0	0
C3M4	2022-06-23	June	Concrete	Middle	0	0
C3M5	2022-06-23	June	Concrete	Middle	0	0
C3T1	2022-06-23	June	Concrete	Top	1.05E+04	9.78E+03
C3T2	2022-06-23	June	Concrete	Top	0	0
C3T3	2022-06-23	June	Concrete	Top	1.60E+03	1.36E+03
C3T4	2022-06-23	June	Concrete	Top	0	0
C3T5	2022-06-23	June	Concrete	Top	0	0
C4B1	2022-07-13	July	Concrete	Bottom	0	0
C4B2	2022-07-13	July	Concrete	Bottom	0	0
C4B3	2022-07-13	July	Concrete	Bottom	0	0
C4B4	2022-07-13	July	Concrete	Bottom	0	0
C4B5	2022-07-13	July	Concrete	Bottom	0	0
C4M1	2022-07-13	July	Concrete	Middle	0	0
C4M2	2022-07-13	July	Concrete	Middle	0	0
C4M3	2022-07-13	July	Concrete	Middle	0	0
C4M4	2022-07-13	July	Concrete	Middle	3.14E+03	3.12E+03
C4M5	2022-07-13	July	Concrete	Middle	0	0
C4T1	2022-07-13	July	Concrete	Top	0	0
C4T2	2022-07-13	July	Concrete	Top	0	0
C4T3	2022-07-13	July	Concrete	Top	0	0

C4T4	2022-07-13	July	Concrete	Top	0	0
C4T5	2022-07-13	July	Concrete	Top	0	0
C5B1	2022-07-20	July	Concrete	Bottom	0	0
C5B2	2022-07-20	July	Concrete	Bottom	0	0
C5B3	2022-07-20	July	Concrete	Bottom	0	0
C5B4	2022-07-20	July	Concrete	Bottom	0	0
C5B5	2022-07-20	July	Concrete	Bottom	0	0
C5M1	2022-07-20	July	Concrete	Middle	0	0
C5M2	2022-07-20	July	Concrete	Middle	0	0
C5M3	2022-07-20	July	Concrete	Middle	0	0
C5M4	2022-07-20	July	Concrete	Middle	0	0
C5M5	2022-07-20	July	Concrete	Middle	0	0
C5T1	2022-07-20	July	Concrete	Top	0	0
C5T2	2022-07-20	July	Concrete	Top	0	0
C5T3	2022-07-20	July	Concrete	Top	0	0
C5T4	2022-07-20	July	Concrete	Top	0	0
C5T5	2022-07-20	July	Concrete	Top	0	0
C6B1	2022-07-27	July	Concrete	Bottom	8.01E+02	7.20E+02
C6B2	2022-07-27	July	Concrete	Bottom	0	0
C6B3	2022-07-27	July	Concrete	Bottom	1.22E+03	1.37E+03
C6B4	2022-07-27	July	Concrete	Bottom	0	0
C6B5	2022-07-27	July	Concrete	Bottom	7.21E+03	7.49E+03
C6M1	2022-07-27	July	Concrete	Middle	1.60E+03	1.65E+03
C6M2	2022-07-27	July	Concrete	Middle	0	0
C6M3	2022-07-27	July	Concrete	Middle	0	0
C6M4	2022-07-27	July	Concrete	Middle	0	0
C6M5	2022-07-27	July	Concrete	Middle	0	0
C6T1	2022-07-27	July	Concrete	Top	9.50E+02	9.62E+02
C6T2	2022-07-27	July	Concrete	Top	0	0
C6T3	2022-07-27	July	Concrete	Top	0	0
C6T4	2022-07-27	July	Concrete	Top	5.99E+03	4.35E+03
C6T5	2022-07-27	July	Concrete	Top	1.26E+03	1.60E+03
C7B1	2022-08-03	August	Concrete	Bottom	4.94E+03	5.37E+03
C7B2	2022-08-03	August	Concrete	Bottom	0	0
C7B3	2022-08-03	August	Concrete	Bottom	0	0
C7B4	2022-08-03	August	Concrete	Bottom	0	0
C7B5	2022-08-03	August	Concrete	Bottom	1.03E+04	7.12E+03
C7M1	2022-08-03	August	Concrete	Middle	0	0
C7M2	2022-08-03	August	Concrete	Middle	1.48E+03	1.89E+03
C7M3	2022-08-03	August	Concrete	Middle	0	0
C7M4	2022-08-03	August	Concrete	Middle	1.27E+03	1.08E+03
C7M5	2022-08-03	August	Concrete	Middle	0	0
C7T1	2022-08-03	August	Concrete	Top	8.20E+03	8.74E+03
C7T2	2022-08-03	August	Concrete	Top	4.24E+03	3.00E+03
C7T3	2022-08-03	August	Concrete	Top	1.52E+03	9.64E+02
C7T4	2022-08-03	August	Concrete	Top	3.18E+03	3.93E+03
C7T5	2022-08-03	August	Concrete	Top	4.94E+03	5.17E+03

C8B1	2022-08-10	August	Concrete	Bottom	2.49E+03	2.07E+03
C8B2	2022-08-10	August	Concrete	Bottom	8.85E+03	1.03E+04
C8B3	2022-08-10	August	Concrete	Bottom	0	0
C8B4	2022-08-10	August	Concrete	Bottom	0	0
C8B5	2022-08-10	August	Concrete	Bottom	0	0
C8M1	2022-08-10	August	Concrete	Middle	4.73E+03	4.29E+03
C8M2	2022-08-10	August	Concrete	Middle	0	0
C8M3	2022-08-10	August	Concrete	Middle	1.09E+03	9.90E+02
C8M4	2022-08-10	August	Concrete	Middle	9.68E+03	7.94E+03
C8M5	2022-08-10	August	Concrete	Middle	1.26E+04	1.18E+04
C8T1	2022-08-10	August	Concrete	Top	1.26E+03	8.07E+02
C8T2	2022-08-10	August	Concrete	Top	1.01E+04	9.08E+03
C8T3	2022-08-10	August	Concrete	Top	1.28E+04	1.15E+04
C8T4	2022-08-10	August	Concrete	Top	2.60E+03	1.09E+03
C8T5	2022-08-10	August	Concrete	Top	1.94E+03	1.87E+03
C9B1	2022-08-17	August	Concrete	Bottom	3.27E+04	3.19E+04
C9B2	2022-08-17	August	Concrete	Bottom	8.36E+03	6.94E+03
C9B3	2022-08-17	August	Concrete	Bottom	5.34E+03	6.15E+03
C9B4	2022-08-17	August	Concrete	Bottom	3.48E+03	2.22E+03
C9B5	2022-08-17	August	Concrete	Bottom	5.27E+03	4.55E+03
C9M1	2022-08-17	August	Concrete	Middle	8.50E+03	5.88E+03
C9M2	2022-08-17	August	Concrete	Middle	4.39E+04	3.54E+04
C9M3	2022-08-17	August	Concrete	Middle	5.49E+03	4.51E+03
C9M4	2022-08-17	August	Concrete	Middle	1.56E+04	1.52E+04
C9M5	2022-08-17	August	Concrete	Middle	2.37E+04	1.96E+04
C9T1	2022-08-17	August	Concrete	Top	1.15E+04	9.90E+03
C9T2	2022-08-17	August	Concrete	Top	8.45E+03	7.49E+03
C9T3	2022-08-17	August	Concrete	Top	1.55E+03	1.39E+03
C9T4	2022-08-17	August	Concrete	Top	2.40E+04	2.03E+04
C9T5	2022-08-17	August	Concrete	Top	1.89E+04	2.00E+04
C10B1	2022-08-24	August	Concrete	Bottom	0	0
C10B2	2022-08-24	August	Concrete	Bottom	2.70E+03	2.75E+03
C10B3	2022-08-24	August	Concrete	Bottom	1.80E+04	1.67E+04
C10B4	2022-08-24	August	Concrete	Bottom	0	0
C10B5	2022-08-24	August	Concrete	Bottom	0	0
C10M1	2022-08-24	August	Concrete	Middle	3.50E+03	2.91E+03
C10M2	2022-08-24	August	Concrete	Middle	1.24E+04	8.89E+03
C10M3	2022-08-24	August	Concrete	Middle	5.85E+03	4.93E+03
C10M4	2022-08-24	August	Concrete	Middle	1.86E+03	1.44E+03
C10M5	2022-08-24	August	Concrete	Middle	1.28E+04	1.17E+04
C10T1	2022-08-24	August	Concrete	Top	2.14E+04	1.84E+04
C10T2	2022-08-24	August	Concrete	Top	2.26E+04	1.61E+04
C10T3	2022-08-24	August	Concrete	Top	1.02E+04	6.23E+03
C10T4	2022-08-24	August	Concrete	Top	0	0
C10T5	2022-08-24	August	Concrete	Top	1.63E+04	1.87E+04
C11B1	2022-09-01	September	Concrete	Bottom	0	0
C11B2	2022-09-01	September	Concrete	Bottom	5.25E+03	6.07E+03

C11B3	2022-09-01	September	Concrete	Bottom	2.11E+03	1.64E+03
C11B4	2022-09-01	September	Concrete	Bottom	1.86E+03	1.27E+03
C11B5	2022-09-01	September	Concrete	Bottom	2.48E+03	3.92E+03
C11M1	2022-09-01	September	Concrete	Middle	0	0
C11M2	2022-09-01	September	Concrete	Middle	2.30E+03	1.13E+03
C11M3	2022-09-01	September	Concrete	Middle	0	0
C11M4	2022-09-01	September	Concrete	Middle	1.40E+03	1.33E+03
C11M5	2022-09-01	September	Concrete	Middle	0	0
C11T1	2022-09-01	September	Concrete	Top	1.06E+04	8.39E+03
C11T2	2022-09-01	September	Concrete	Top	5.05E+03	4.80E+03
C11T3	2022-09-01	September	Concrete	Top	5.32E+03	4.07E+03
C11T4	2022-09-01	September	Concrete	Top	0	0
C11T5	2022-09-01	September	Concrete	Top	1.01E+04	7.52E+03
C12B1	2022-09-15	September	Concrete	Bottom	3.02E+04	1.94E+04
C12B2	2022-09-15	September	Concrete	Bottom	2.99E+03	3.21E+03
C12B3	2022-09-15	September	Concrete	Bottom	5.82E+03	5.26E+03
C12B4	2022-09-15	September	Concrete	Bottom	9.30E+03	8.18E+03
C12B5	2022-09-15	September	Concrete	Bottom	3.05E+03	3.40E+03
C12M1	2022-09-15	September	Concrete	Middle	4.84E+03	2.56E+03
C12M2	2022-09-15	September	Concrete	Middle	2.72E+03	2.29E+03
C12M3	2022-09-15	September	Concrete	Middle	1.19E+03	1.36E+03
C12M4	2022-09-15	September	Concrete	Middle	1.42E+03	1.17E+03
C12M5	2022-09-15	September	Concrete	Middle	7.43E+03	5.32E+03
C12T1	2022-09-15	September	Concrete	Top	7.57E+03	6.49E+03
C12T2	2022-09-15	September	Concrete	Top	6.41E+03	5.94E+03
C12T3	2022-09-15	September	Concrete	Top	4.12E+03	3.67E+03
C12T4	2022-09-15	September	Concrete	Top	1.43E+04	1.19E+04
C12T5	2022-09-15	September	Concrete	Top	8.53E+04	8.48E+04
C13B1	2022-10-05	October	Concrete	Bottom	0	0
C13B2	2022-10-05	October	Concrete	Bottom	0	0
C13B3	2022-10-05	October	Concrete	Bottom	0	0
C13B4	2022-10-05	October	Concrete	Bottom	0	0
C13B5	2022-10-05	October	Concrete	Bottom	6.62E+03	4.77E+03
C13M1	2022-10-05	October	Concrete	Middle	0	0
C13M2	2022-10-05	October	Concrete	Middle	0	0
C13M3	2022-10-05	October	Concrete	Middle	0	0
C13M4	2022-10-05	October	Concrete	Middle	0	0
C13M5	2022-10-05	October	Concrete	Middle	0	0
C13T1	2022-10-05	October	Concrete	Top	0	0
C13T2	2022-10-05	October	Concrete	Top	0	0
C13T3	2022-10-05	October	Concrete	Top	4.45E+03	3.56E+03
C13T4	2022-10-05	October	Concrete	Top	5.86E+03	5.12E+03
C13T5	2022-10-05	October	Concrete	Top	0	0
C14B1	2022-10-26	October	Concrete	Bottom	0	0
C14B2	2022-10-26	October	Concrete	Bottom	1.50E+03	1.19E+03
C14B3	2022-10-26	October	Concrete	Bottom	4.39E+03	2.77E+03
C14B4	2022-10-26	October	Concrete	Bottom	0	0

C14B5	2022-10-26	October	Concrete	Bottom	3.80E+03	4.46E+03
C14M1	2022-10-26	October	Concrete	Middle	0	0
C14M2	2022-10-26	October	Concrete	Middle	0	0
C14M3	2022-10-26	October	Concrete	Middle	0	0
C14M4	2022-10-26	October	Concrete	Middle	2.57E+03	1.80E+03
C14M5	2022-10-26	October	Concrete	Middle	0	0
C14T1	2022-10-26	October	Concrete	Top	0	0
C14T2	2022-10-26	October	Concrete	Top	0	0
C14T3	2022-10-26	October	Concrete	Top	0	0
C14T4	2022-10-26	October	Concrete	Top	0	0
C14T5	2022-10-26	October	Concrete	Top	0	0
C15B1	2022-11-15	November	Concrete	Bottom	0	0
C15B2	2022-11-15	November	Concrete	Bottom	3.17E+03	1.66E+03
C15B3	2022-11-15	November	Concrete	Bottom	4.27E+04	2.31E+04
C15B4	2022-11-15	November	Concrete	Bottom	0	0
C15B5	2022-11-15	November	Concrete	Bottom	0	0
C15M1	2022-11-15	November	Concrete	Middle	0	0
C15M2	2022-11-15	November	Concrete	Middle	0	0
C15M3	2022-11-15	November	Concrete	Middle	0	0
C15M4	2022-11-15	November	Concrete	Middle	0	0
C15M5	2022-11-15	November	Concrete	Middle	0	0
C15T1	2022-11-15	November	Concrete	Top	0	0
C15T2	2022-11-15	November	Concrete	Top	0	0
C15T3	2022-11-15	November	Concrete	Top	0	0
C15T4	2022-11-15	November	Concrete	Top	0	0
C15T5	2022-11-15	November	Concrete	Top	0	0
